# Cerebrospinal fluid proteomics in meningitis patients with reactivated varicella zoster virus

**DOI:** 10.1002/iid3.1038

**Published:** 2023-10-04

**Authors:** Huili Liu, Jun Wang, Yan Zhang, Jing Gu, Yu Wang, Yongxing Yan, Dongli Pan, Zeyu Sun

**Affiliations:** ^1^ Department of Neurology Hangzhou Third People's Hospital Hangzhou Zhejiang China; ^2^ Department of Medical Microbiology and Parasitology Zhejiang University School of Medicine Hangzhou Zhejiang China; ^3^ State key Laboratory for Diagnosis and Treatment of Infectious Diseases, National Clinical Research Center for Infectious Diseases, Collaborative Innovation Center for Diagnosis and Treatment of Infectious Disease, The First Affiliated Hospital Zhejiang University School of Medicine Hangzhou Zhejiang China

**Keywords:** meningitis, proteomics, cerebrospinal fluid, varicella‐zoster virus

## Abstract

**Objective:**

This study investigated the proteomic characteristics of cerebrospinal fluid (CSF) in patients with varicella zoster virus (VZV) meningitis to understanding the pathogenesis of central nervous system (CNS) infection by reactivated VZV.

**Method:**

We used data‐independent acquisition model to analyze the CSF proteomic differences of 28 patients with VZV meningitis and 11 herpes zoster (HZ) patients. According to the clinical manifestations at discharge, 28 VZV meningitis patients were divided into favorable outcome group and unfavorable outcome (UO) group and their differences in CSF proteome were also analyzed.

**Results:**

Compared with the HZ group, the proteins (CXCL10, ELANE, IL‐1RN, MPO, PRTN3, etc.) related to inflammation and immune cell activation were significantly upregulated in the VZV meningitis group (*p* < .01). The protein related to the nerve function and energy metabolism (CKMT1B, SLITRK3, Synaptotagmin‐3, KIF5B, etc.) were significantly downregulated (*p* < .05). The levels of a pro‐inflammatory factor, IL‐18, in CSF were significantly higher in patients in the UO group as compared to patients with favorable prognosis (*p* < .05).

**Conclusion:**

Inflammatory immune response is an important pathophysiological mechanism of CNS infection by VZV, and the CSF IL‐18 levels might be a potential prognostic indicator of the outcomes of VZV meningitis.

## Graphical abstract

Inflammatory immune response is an important pathophysiological mechanism of central nervous system infection by varicella zoster virus (VZV), and the cerebrospinal fluid IL‐18 levels might be a potential prognostic indicator of the outcomes of VZV meningitis.

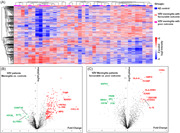

## 1 INTRODUCTION

Varicella zoster virus (VZV) is a herpesvirus belonging to the genus Varicellovirus that primarily attacks human neurons. After infection, VZV stays latent in trigeminal ganglia or dorsal root ganglia. When patient's immune system is compromised, the latent VZV can be reactivated and leads to regional rash and severe pain, known as herpes zoster (HZ), which can also affect peripheral nervous system. The most common complication of HZ is postherpetic neuralgia (PHN) causing intractable chronic pain. HZ can also cause intracranial infection,^1^, ^2^, ^3^ which brings substantial physical, psychological, and economical burdens to the patients. VZV‐related central nervous system (CNS) infection can even be fatal with a mortality rate that can reach 5%.^4^

VZV meningitis is one of the common complications of CNS infection caused by reactivated VZV. The pathological mechanism of VZV meningitis is multifactorial. Direct viral infection, inflammation, virus‐induced hypercoagulability, vasculitis, and so on have been implicated,^5^, ^6^, ^7^ but its exact mechanism remains elusive.

Studying responses to infections at the proteome level facilitates early diagnosis, risk stratification, and prediction of outcomes, and can help to understand the pathophysiological mechanisms. But to date, few studies have been conducted on CSF proteomics in patients with VZV meningitis.^8^, ^9^, ^10^, ^11^, ^12^ In this study, the highly sensitive and reproducible data‐independent acquisition (DIA) based proteomics technology was employed to evaluate the differences in protein levels in CSF of patients with VZV meningitis and HZ, aiming to identify protein markers that are specifically dysregulated in patients with VZV meningitis and to improve our understanding of the pathological mechanism of VZV CNS infection.

## 2 METHODS

### 2.1 Patients information

Twenty‐eight patients with VZV meningitis hospitalized in the Department of Neurology, Hangzhou Third People's Hospital from June 2021 to November 2022 were selected, including 21 males and seven females, with an average age of 56.5 ± 16.3 years. Eleven patients with HZ hospitalized during the same period were selected as the control group, including three males and eight females. The average age was 54.5 ± 16.3 years. The diagnosis, treatment, and follow‐up records of patients with HZ and VZV meningitis were obtained through the hospital case information system. The contents of the records include genders, ages, sites of HZ, disease courses, concomitant diseases, clinical symptoms, results of routine and biochemical examinations of CSF, lengths of stay, treatment situations, and so on.

### 2.2 Inclusion and exclusion criteria

Patients with shingles within 2 weeks of onset were selected. Shingles was determined by dermatologists according to the diagnostic criteria.^13^ The diagnosis of VZV meningitis is as follows: (1) HZ within 2 weeks of onset; (2) Any two of the symptoms/signs of meningeal irritation such as fever, headache, nausea, vomiting, and neck stiffness; (3) No symptom of acute brain parenchymal injury; (4) CSF leukocyte count >5 × 10^6^/L or protein content >0.45 g/L or positive VZV DNA. Exclusion criteria: (1) Patients ≤14 years; (2) CSF test confirmed bacterial, fungal, and other microbial infections; (3) Patients with incomplete clinical data; (4) HIV‐infected patients; (5) Patients with mental illness or severe dementia who could not accurately express clinical symptoms.

### 2.3 Outcomes of VZV meningitis patients

The outcomes of patients with VZV meningitis were judged by their conditions at the time of discharge. The favorable outcome (FO) group includes those patients that had no symptoms of discomfort upon discharge. The unfavorable outcome (UO) was defined as death, severe disability, or any neurological sequelae affecting the patient's daily life at the time of discharge.

### 2.4 CSF sample collection

All patients underwent lumbar puncture within 48 h of admission, and 2 mL of cerebrospinal fluid (CSF) was reserved for routine examination (leukocyte count, glucose, chlorine, total protein, adenosine deaminase, lactate dehydrogenase and proteomic analysis. The detailed steps are as follows.

### 2.5 Protein trypsin digestion

According to the protein concentration of each sample, 25 μg of protein was reduced by 5 mM dithiothreitol for 45 min followed by alkylation by 12 mM iodoacetamide in the dark for 40 min. Protein was then precipitated by acetone and digested by 0.5 μg trypsin (V5111, Promega) in 100 mM ammonium bicarbonate at 37°C for 14 h. The peptides were acidified with 0.1% formic acid (FA) and desalted by hydrophilic‐lipophilic‐balanced plates (Waters) before nanoLC‐MS/MS analysis.

### 2.6 LC‐MS/MS DIA analysis

Peptides were separated by the U3000 UPLC nano liquid phase (ThermoFisher) system equipped with a self‐packed column (75 μm × 250 mm, 1.9 μm Reprosil‐Pur C18 beads) (Dr. Maisch) with a flow rate of 450 nL/min. Mobile phase A: 0.1% FA, B: 0.1% FA dissolved in 98% acetonitrile. The 120‐min gradient is set as follows: 5 min 3%–8%B; 85 min 8%–24%B; 20 min 24%–38%B; 5 min 38%–80%B. It was then held at 80% B for 5 min until the next injection. The peptides were analyzed by Exploris 480 mass spectrometry working on the DIA mode that collects a full MS survey scan from 400 to 1000 Th at the resolution of 120,000 full‐width at half‐maximum (FWHM) (at m/z 200 Th) with automatic gain control (AGC) set to 100%, followed by MS2 spectra with 75 isolation windows of 8 Th with an overlap of 1 Th. Precursors co‐isolated in each window were fragmented by higher‐energy collision dissociation with normalized collision energy set to 30%. All MS2 spectra were acquired with 30,000 FWHM resolution with AGC set to standard.

### 2.7 DIA data analysis

The collected RAW files were loaded into the DIA‐NN v1.8 for spectral‐library‐free search against the human UniProt database supplemented with VZV and common contamination sequences. Peptide length was restricted to 7–30 amino acids and precursor charge was limited to <5. Other search parameters were set as follows: oxidation (M) and carbamoyl (C) were set as variable and fixed modification, respectively; protease was set to trypsin and a maximum of two missed cut sites were allowed, and the mass error was set to 7 ppm. The results were filtered by PSM and protein level 1% false discovery rate. For quantitative analysis, relative protein intensity in each sample was log2 transformed. Student's‐*t* test (*p* < .05) and fold‐change (≥1 or ≤−1) was used to select differentially expressed proteins.

### 2.8 Statistical analysis

SPSS 20.0 software was used for data processing and statistical analysis. The measurement data of normal distribution were expressed as mean ± standard deviation. T test was used to compare the mean of two samples. Data with nonnormal distribution were described by median (interquartile distance) {M (Q1–Q3)}, and comparison between the two groups was conducted by Mann–Whitney U test. Count data were expressed by frequency and percentage, and comparison between groups was performed by Chi‐square test or Fisher's exact test. *p* < .05 was considered to be statistically significant.

## 3 RESULTS

### 3.1 Comparison of general characteristics between HZ control and VZV meningitis groups

There was no difference in ages and concomitant diseases between the HZ control (without CNS infection) and VZV meningitis groups, but there were more males than females with VZV meningitis, the difference was statistically significant (*p* < .05).

The zoster site of VZV meningitis occurred in the head and face in 20 cases (71.4%), followed by the chest and back in four cases (14.3%), neck (10.7%) in three cases, waist (3.6%) in one case. In the HZ group, zoster was mainly distributed in the head and face in six cases (54.5%), followed by chest and back in five cases (45.5%) and neck in two cases (18.2%). Among the 28 cases of VZV meningitis, there were 11 cases (39.3%) with headache, 14 cases (50.0%) with fever, five cases (17.9%) with vertigo, and RHS (Ramsay Hunt Syndrome) five cases (17.9%). In the HZ group, there were six cases (54.5%) with headache and one case (9.1%) with fever. There were no significant differences in the zosters site and clinical symptoms between the HZ control and VZV meningitis groups (*p* > .05). The data are shown in Table 1.

**Table 1:** Baseline characteristics of patients with herpes zoster and VZV meningitis.

	Herpes zoster group (*n* = 11)	VZV meningitis group (*n* = 28)	*p* Value
Age (x ± s) (years)	54.5 ± 16.3	56.5 ± 16.3	.726
Gender			
Male	3	21	.006
Female	8	7	
Concomitant disease			
Coronary heart disease	0	1	
COPD	2	1	
Hypertension	3	10	
Immune disease	0	1	
Immunodrug use	0	1	
Stroke	0	1	
Diabetes	2	3	
Chronic kidney disease	1	2	
Chronic liver disease	1	3	
Tumor	0	1	
Herpetic site			
Head and face	6	20	
Neck	2	3	
Thoracolumbra	5	4	
Waist	0	1	
Limbs	0	2	
Clinical symptom (case)			
Headache	6	11	
Fever	1	14	
Vertigo	0	5	
RHS	0	5	
Length of stay (days)	10.8 ± 3.3	15.2 ± 3.7	.002
Days of antiviral treatment (days)	8.1 ± 3.9	13.9 ± 2.8	.000
Glucocorticoid therapy (case. %)	6 (54.5%)	26 (92.9%)	.005
Time between herpes onset and antiviral treatment (median [Q1–Q3]) (days)	2 (1, 4)	3 (2, 4.5)	.938
CSF testing			
Leukocyte count (median [Q1–Q3]) (×10^6^/L)	2 (2, 3)	27.5 (3, 45)	.000
Glucose (mmol/L)	4.0 ± 1.4	3.8 ± 0.8	.606
Chlorine (mmol/L)	127.4 ± 4.6	122.6 ± 5.8	.019
Protein (mg/dL)	40.7 ± 16.7	71.7 ± 32.7	.005
ADA (U/L)	1.3 ± 0.8	1.4 ± 1.1	.683
LDH (U/L)	24.2 ± 7.0	32.3 ± 14.1	.076

Abbreviations: ADA, adenosine deaminase; COPD, chronic obstructive pulmonary disease; CSF, cerebrospinal fluid; LDH, lactate dehydrogenase; RHS, Ramsay Hunt Syndrome; VZV, varicella zoster virus.

### 3.2 Treatment

All patients received intravenous antiviral therapy and intravenous glucocorticoid for 3–5 days (40–80 mg/d Methylprednisolone). The mean hospital stay in the VZV meningitis group was 15.2 ± 3.7 days, which was significantly longer than that in the HZ group (10.8 ± 3.3 days) (*p* < .05). The number of days of antiviral therapy in the VZV meningitis group was 13.9 ± 2.8 days, which was also longer than that in HZ group 8.1 ± 3.9 days. The proportion of patients in the VZV meningitis group receiving glucocorticoid therapy (26 cases, 92.9%) was significantly higher than that in the HZ group (six cases, 54.5%). The data are shown in Table 1.

### 3.3 CSF analysis

**Figure 1:**
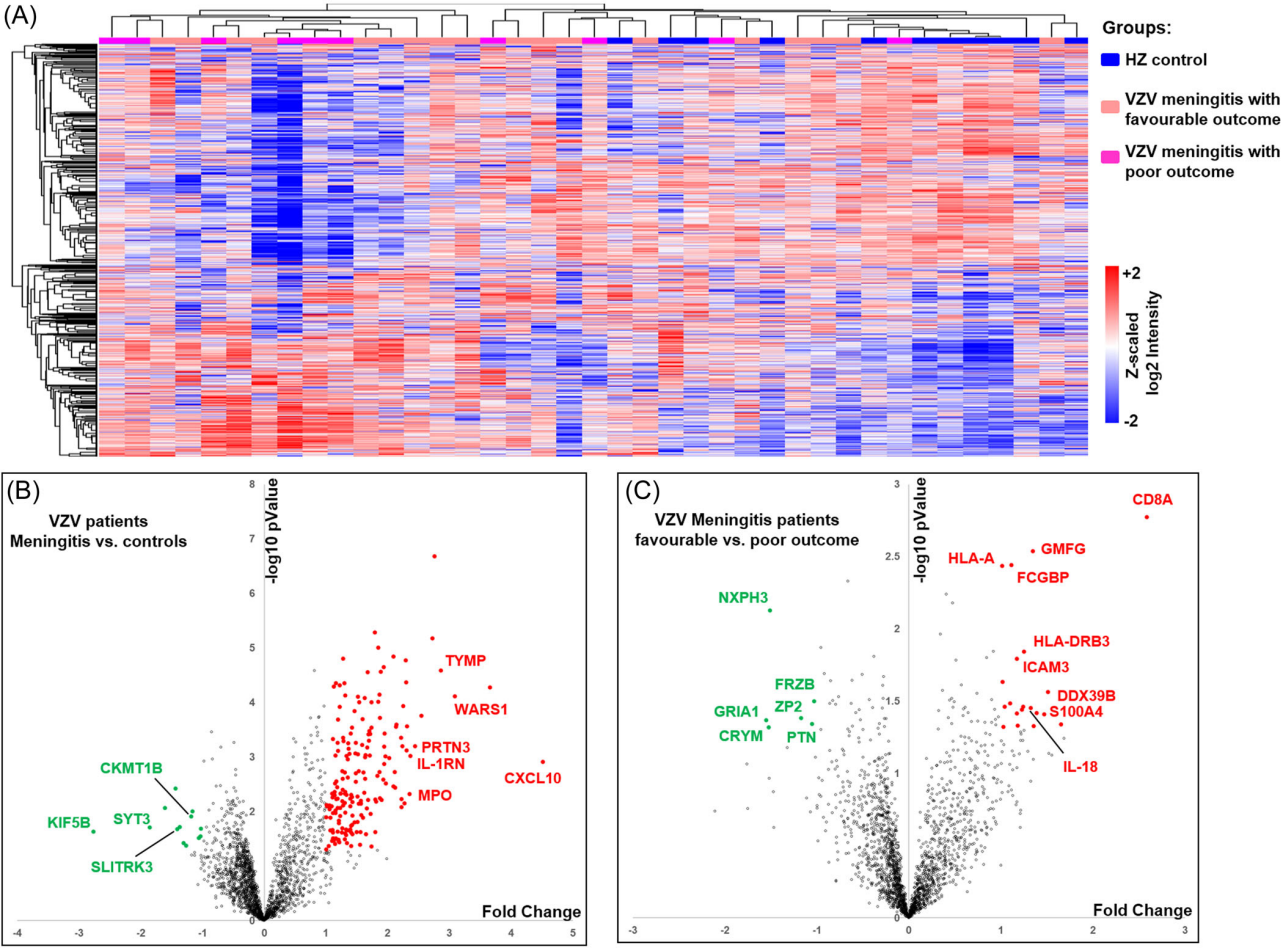
Summary of a proteomics survey of cerebrospinal fluid samples from varicella zoster virus (VZV) meningitis and herpes zoster patients. Clustering analysis represented in heatmap of all protein levels in all samples (A). Quantitation data in Log2 scale were transformed into Z‐score by rows. Sample and protein distances were calculated by Spearman correlation. Red/blue shade correlates with relative high/low protein abundance across each row. Volcano plot showing differentially expressed proteins between VZV‐meningitis and controls (B), and between VZV‐meningitis of favorable and poor outcomes (C).

**Figure 2:**
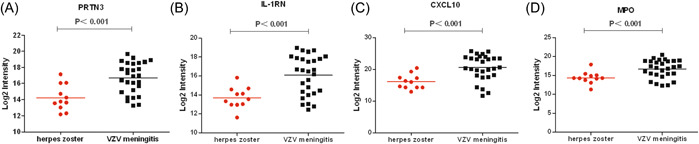
Differentially expressed proteins related to the inflammation and immune cell activation were shown between varicella zoster virus meningitis and herpes zoster groups. (A, PRTN3; B, IL‐1RN; C, CXCL10; D, MPO).

**Figure 3:**
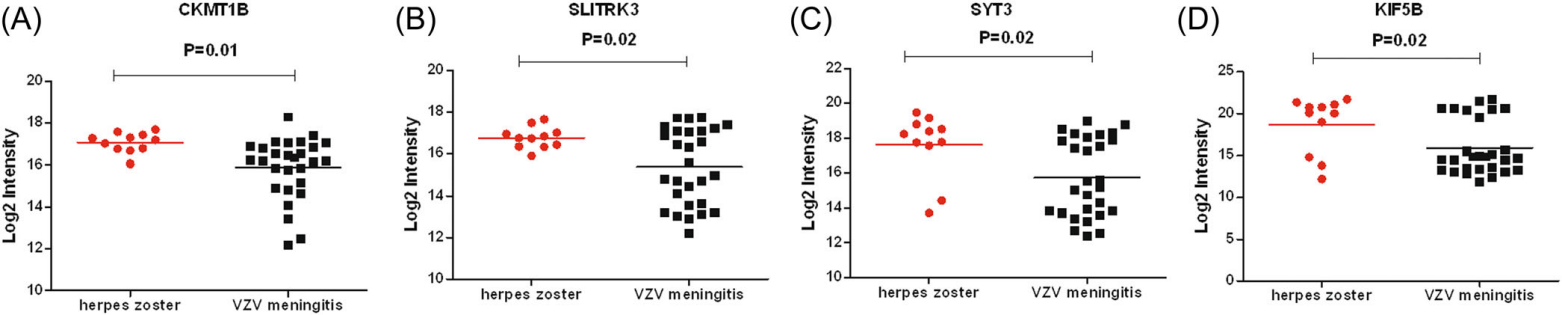
Differentially expressed proteins related to the nerve function and energy metabolism were shown between varicella zoster virus meningitis and herpes zoster groups. (A, CKMT1B; B, SLITRK3; C, SYT3; D, KIF5B).

The average leukocyte count (27.5 × 10^6^/L, protein content (71.7 ± 32.7) mg/dL, chlorine content (122.6 ± 5.8) mmol/L in the CSF of the VZV meningitis group was significantly higher than that of the HZ group, (*p* < .05, Table 1). A total of 2502 proteins were identified in CSF proteomics (Figure 1). Compared with the HZ group, the expression of neutrophil‐activating proteins (ELANE, MPO, PRTN3), and proteins reflecting inflammation and immune cell activation (CXCL10, WARS1, IL‐1RN, etc.) was significantly increased (*p* < .01), while the expression of proteins related to nerve function and energy metabolism (CKMT1B, SLITRK3, Synaptotagmin‐3 [SYT3], KIF5B, etc.) was significantly decreased (*p* < .05) (Figures 1, 2, 3).

### 3.4 Proteomics analysis of VZV meningitis patients with different outcome

According to prognostic assessment criteria, 18 patients (64.3%) with VZV meningitis had FO at discharge, while 10 patients (35.7%) had UO. Among them, the proportion of female patients with UO was higher, with statistical difference (*p* < .05). The interval between the onset of neurological symptoms to antiviral therapy in the UO group was significantly longer than that in the FO group (*p* < .05) Table 2.

**Table 2:** Characteristics of VZV meningitis patients with different outcomes.

	FO group (*n* = 18)	UO (*n* = 10)	*p* Value
Age (x ± s) (years)	56.7 ± 18.6	56.2 ± 12.1	.944
Gender			
Male	16	5	.023
Female	2	5	
Length of stay (days)	14.9 ± 3.2	15.8 ± 4.7	.545
Days of antiviral treatment (days)	13.6 ± 2.5	14.4 ± 4.7	.461
Glucocorticoid therapy (case. %)	16 (88.9%)	10 (100%)	.862
Time between herpes onset and antiviral treatment (median [Q1–Q3]) (days)	4.5 (3, 5)	7 (3, 11.75)	.041
CSF testing			
Leukocyte count (median [Q1–Q3]) (×10^6^/L)	18 (2, 45)	35 (5.25, 57.5)	.442
Glucose (mmol/L)	4.0 ± 0.8	3.6 ± 0.8	.243
Chlorine (mmol/L)	122.1 ± 4.9	123.5 ± 7.4	.539
Protein (mg/dL)	73.4 ± 33.1	68.7 ± 33.6	.762
ADA (U/L)	1.5 ± 1.1	1.2 ± 1.1	.474
LDH (U/L)	31.4 ± 7.9	34.0 ± 21.7	.646

Abbreviations: ADA, adenosine deaminase; CSF, cerebrospinal fluid; FO, favorable outcome; LDH, lactate dehydrogenase; UO, unfavorable outcome; VZV, varicella zoster virus.

There were no differences in ages, lengths of stay, days of antiviral therapy, proportions of glucocorticoid therapy, CSF leukocyte counts, protein, and glucose contents between the two groups (*p* > .05).

**Figure 4:**
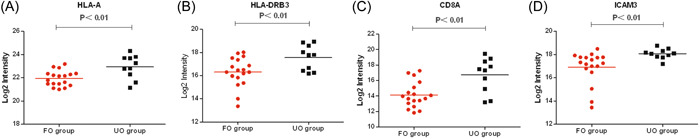
Differentially expressed proteins related to the inflammation and immune cell activation were shown between favorable outcome and unfavorable outcome groups. (A, HLA‐A; B, HLA‐DRB3; C, CD8A; D, ICAM3).

**Figure 5:**
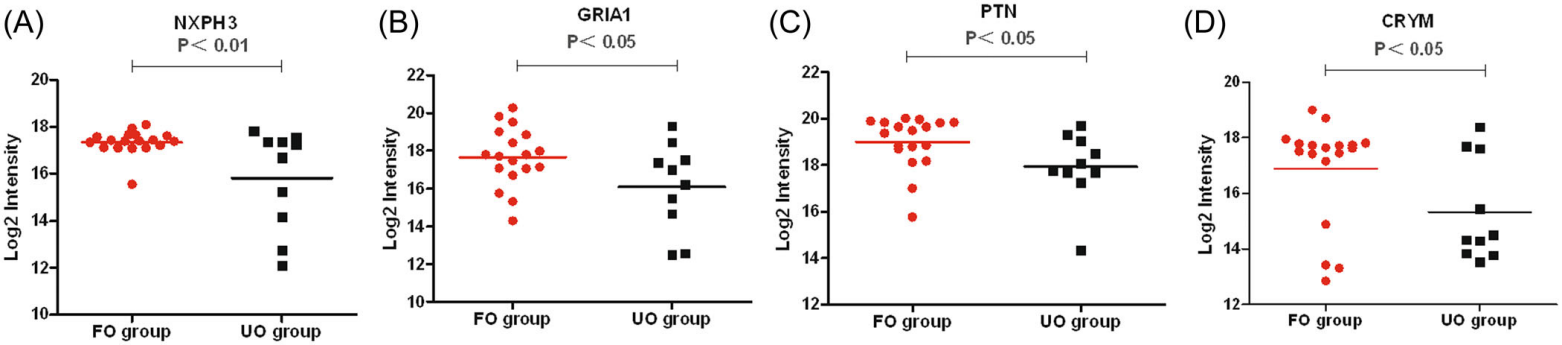
Differentially expressed proteins related to the neurodevelopmental homeostasis and nerve signal transduction were shown between favorable outcome and unfavorable outcome groups. (A, NXPH3; B, GRIA1; C, PTN; D, CRYM).

**Figure 6:**
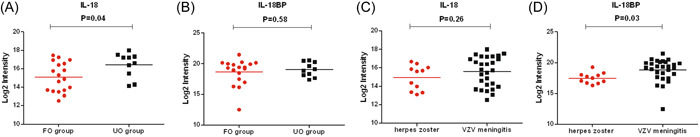
IL‐18 and IL‐18BP expression in different groups. Compared with the favorable outcome group, (A) IL‐18 was significantly upregulated in varicella zoster virus (VZV) meningitis patients in the unfavorable outcome group (*p* = .04), (B) but IL‐18BP expression was unchanged (*p* = .58), (D) The levels of IL‐18BP in cerebrospinal fluid of VZV meningitis patients were significantly higher than those of herpes zoster (HZ) control group (*p* = .03), (C) but the IL‐18 levels showed no difference between VZV meningitis and HZ control groups (*p* = .26).

Proteomic analysis showed that 50 proteins were upregulated and 35 proteins were downregulated in the CSF of VZV meningitis patients in the UO group compared with the FO group. The levels of proteins that reflect inflammatory and immune responses (IL‐18, ICAM3, CD8A, GMFG, HLA‐A, HLA‐DRB3, etc.) was significantly increased (*p* < .05) (Figure 4). while those of proteins related to neurodevelopmental homeostasis and nerve signal transduction (NXPH3, GRIA1, PTN, CRYM etc.) were significantly decreased (*p* < .05) (Figure 5). There was no statistical difference in the levels of IL‐18BP, which act as an antagonist of pro‐inflammatory factor IL‐18 (*p* > .05) (Figure 5). Interestingly, the levels of IL‐18BP, but not IL‐18, in the CSF of the VZV meningitis group were significantly higher than that of the HZ control group (Figure 6).

## 4 DISCUSSION

VZV lurks in the peripheral and CNSs, and can be reactivated when the host immune function is repressed.^14^ Reactivated VZV can cause a wide range of CNS manifestations, such as encephalitis, meningitis, RHS and vasculitis with stroke‐like symptom.^1^, ^2^, ^3^ With increased attention of clinicians to VZV‐related CNS infection, increasing cases of VZV meningitis and encephalitis in hosts with competent immune functions have also been identified.^15^, ^16^, ^17^ Most of the VZV meningitis patients included in this study presented with fever, headache, meningeal irritation, and increased leukocyte counts and protein contents in CSF. Among them, VZV DNA (+) in CSF was found in 17 case, and zosters developed in all patients within 2 weeks of onset, which ensured the accuracy of diagnosis of VZV meningitis. Compared with the control HZ patients without CNS infection, the clinical characteristics of VZV meningitis was similar to previous reported.^5^, ^6^

VZV is one of the most common pathogenic herpesviruses in CNS infection,^18^ and VZV causes aseptic meningitis in 5%–27% of cases.^19^, ^20^ But its exact pathological mechanism remains to be further explored. The host response to the invasion of viral pathogens into the CNS can results in specific proteomic signatures in CSF. Previous studies have found increased levels of inflammatory cytokines and chemokines in the CSF of patients with viral meningitis, such as IL‐6, IL‐1b, TNF‐α, IL‐10, CXC family chemokines and growth factors.^21^, ^22^ Lind et al.^10^ found that high levels of CXCL9 and CXCL10 in the CSF of patients with herpes simplex encephalitis/meningitis, and the concentrations in the CSF were higher than those in the serum. The study on aseptic meningitis caused by nonpoliomyelitis enterovirus infection in children showed that the levels of CXCL10 and CXCL11 in CSF were higher than those in normal children, and CXCL10 levels may have high differential value to identify nonpolio aseptic aseptic meningitis.^23^ Other studies have revealed that CXCL10 protein in CSF was most significantly upregulated in children with enteroviruses encephalitis.^24^, ^25^ Another meta‐analysis showed elevated levels of IL‐1β in CSF in aseptic meningitis patients.^26^ This suggests that virus entered the CNS and induced an immune response. Previous studies on CSF protein in viral encephalitis often selected some specific proteins, so they could not reveal the changes of CSF protein content in a panoramic manner.

Our study showed that inflammation is an important mechanism in the occurrence and development of VZV meningitis. In this study, we used DIA technology to detect 39 CSF samples (28 cases of VZV meningitis, 11 cases of control HZ without CNS infection) and identified 2502 proteins in the CSF. We found that the expression of neutrophil‐activating proteins (ELANE, MPO, PRTN3) and proteins reflecting inflammation and immune cell activation (CXCL10, WARS1, IL‐1RN, etc.) were significantly upregulated, while those related to nerve function and energy metabolism (CKMT1B, SLITRK3, SYT3, KIF5B, etc.) were downregulated.

Normally, leukocyte entrance into the CNS is limited by the blood–brain barrier and/or by the blood‐CSF barrier. Our study found that the CSF leukocytes count and neutrophil activation markers, ELANE, MPO, and PRTN3 were most increased in the VZV meningitis group. ELANE is a serine protease released by neutrophils that can cause harmful inflammatory responses. Sun et al.^27^ found that the levels of serum neutrophilic elastase in patients with influenza encephalopathy were significantly higher than those in patients with uncomplicated influenza, suggesting that brain endothelial injury during the development of influenza was mediated by neutrophilic elastase. A traumatic experiment in a mouse model also suggests that inhibiting the activity of neutrophil elastase can reduce secondary blood–brain barrier damage.^28^ Rugemalira et al. observed a high MPO activation in CSF in children with bacterial meningitis as compared to those of nonbacterial meningitis and also found that activated MPO can be used as a biomarker for grading inflammation severity.^29^ Significant changes in the expression of PRTN3, MPO, and ELANE proteins are very important in the early stage of infection and are significantly related to the severity of the disease. Regulation of PRTN3 and MPO may alleviate the symptoms of SARS‐CoV‐2 by promoting anti‐inflammatory response.^30^ The increased levels of ELANE, MPO, and PRTN3 in CSF of patients with VZV meningitis suggest that neutrophil‐mediated inflammation plays an important role in the pathogenesis of CNS infection of VZV. CXCL10 mainly promotes the activation, maturation, and migration of T cells, monocytes, and macrophages, promoting inflammatory progression by causing leukocytes infiltration to the site of inflammation. CXCL10/CXCR3 interaction plays a key role in promoting the accumulation of immunoactive cells, for example, CD8 effector T cells, in CNS.^31^ Our CSF proteomics data suggested that other inflammatory regulatory proteins such as IL1RN, LILRB2, and IL18BP were also significantly elevated. IL1RN is very similar to IL‐1 in structure and can bind to the IL‐1 receptor competitively with IL‐I, thus blocking the effect of IL‐1. However, the enhanced activity of IL‐1 can promote the secretion of IL‐l8, IL‐2, IL‐6, and TNF‐γ and thus stimulate the phagocytosis of neutrophils to clear the infected virus and form the immune complex. Autopsy assessment by Suzuki et al. revealed that VZV encephalitis is hallmarked by the presence of multinucleated cells with ground glass and irregular chromatin edges under the microscope. Neuropathological examination of histological sections of the cerebral cortex revealed multiple inflammatory foci in the viral space, mainly neutrophils and histiocytic infiltration, and a large number of white blood cells, mainly neutrophils, were observed in the cerebral cortical vessels,^32^ further suggesting the prominent role of neutrophil‐mediated inflammatory response in CNS infection of VZV.

This study showed that the levels of proteins related to nerve function and energy metabolism decreased significantly (CKMT1B, KIF5B, SLITRK3, SYT3, etc.). In tissues with high energy requirements, including kidney, brain, testis, placenta, and sperm, CKMT1B expression was significantly negatively correlated with the infiltration levels of B cells, CD8 + T cells, CD4 + T cells, macrophages, neutrophils, and dendritic cells.^33^ KIF5B, SLITRK3, and SYT3 can affect the electrical activity of nerves. The kinesin heavy chain KIF5B exists in the dendritic spine and is involved in axon protein transport, mitochondrial biosynthesis, neural metabolism, and other functions, providing proteins and lipids for neurons so that neurons can effectively cope with oxidative stress.^34^, ^35^ It was further found that KIF5B was involved in dendrite transport, synaptic plasticity, and memory defects.^36^ SLITRK3 is a synaptic formation molecule that selectively inhibits synaptic development.^37^ SYT3 is a transmembrane synaptic binding protein that acts as a calcium receptor in neurons to trigger vesicle fusion and control the rapid fusion of vesicles and membranes. SYT3 can be expressed throughout the brain and may be expressed in T cells to participate in T cell migration.^38^, ^39^ Our results are consistent with literature reports^40^, ^41^ that metabolic disorders are associated with herpesvirus infection. In addition, one study also showed signs of low metabolism in parts of the brain in patients with herpes simplex encephalitis compared to normal controls.^42^

Currently, HSV‐1, HSV‐2, and VZV are known to be common agents of CNS infection in adults and lead to significant neurological morbidity and mortality. Clinical symptoms and disease outcome depend on the specific infectious agent and the immune status of the host.^43^ Our study found that 64.3% of VZV meningitis patients had good outcomes at discharge and 35.7% had poor outcomes and that the longer the interval between the onset of HZ and antiviral treatment, the worse the outcome for VZV meningitis patients. These results are consistent with the results by Persson A et al. that showed that 50% of patients with VZV meningitis developed neurological sequelae at 1‐month follow‐up after discharge.^44^ This study also found that female patients had poor outcomes at discharge, which may be related to the increased incidence of postherpetic neuralgia in female patients. There were also more patients in VZV meningitis group receiving glucocorticoid therapy than that in HZ group, and glucocorticoid therapy could suppress the immune response, further suggesting the presence of intracranial immune response. In this study, proteomics showed that the contents of CD8A (i.e., CD8 alpha chain), HLA‐related proteins, IL‐18, S100A4, and ICAM3 were significantly increased in VZV meningitis patients with poor prognosis. As important members of T cell toxicity pathway genes, CD8 are expressed in cytotoxic T cells and are critical for T cell development through binding to major histocompatibility complex Class I (MHC I) proteins. As a marker of immune cells, The CD8A is a member of T cytotoxic pathway‐related genes and encodes the CD8 antigen that is a cell surface glycoprotein found on most cytotoxic T cells. CD8 + T cells are important not only for the control of acute CNS infection, but also for the maintenance of HSV latent infection in infected ganglia.^45^ The postmortem neuropathological results of 10 patients with COVID‐19 were analyzed. Immunohistochemical staining showed that perivascular inflammatory infiltration was mainly composed of CD8‐positive T cells and CD68‐positive macrophages.^46^ Previous study has found that human major histocompatibility leukocyte antigen (MHC) or human histocompatibility leukocyte antigen (HLA), namely haplotype HLA‐A*3303, HLA‐B*4403, DRB1*1302, is associated with pain after HZ by studying the mouse model of pain after HZ infection. It is believed that HLA‐A*3303, HLA‐B*4403, DRB1*1302, or HLA haplotype's excessive immune response to VZV antigen is one of the important risk factors for human PHN pathogenesis, and MHC‐induced immune response may affect the pathogenesis and prognosis of human PHN.^47^ The upregulated expression of HLA proteins in the CSF of VZV meningitis patients suggests the presence of sustained and more severe inflammation and immune response in the intracranial brain, leading to a worse prognosis for these patients. Compared with the HZ group, proteomics showed no difference in the level of IL18 in CSF of VZV meningitis patients. However, in poor‐outcome patients with VZV meningitis, IL18 levels were significantly elevated. Further analysis showed that the corresponding antagonistic IL18BP molecule increased in VZV meningitis than HZ patients, but did not increase significantly in the poor‐outcome group relative to the good‐outcome patients, suggesting that the IL18 inflammatory pathway may be more significant in patients with poor outcome and the expression level of IL‐18 protein in CSF may be a potential indicator for the outcome of patients with VZV meningitis.

As an ideal specimen for the CNS studies, CSF can accurately and comprehensively reflect the proteomic information of brain diseases, and proteomics can identify potential driving factors and improve our understanding of the pathogenesis of VZV reactivation. Nonetheless, our study did came with limitations. First, the sample size is relatively small, particularly on the control group of 11 HZ patients, which could affect the power to reveal proteomic differences. Second, the time points for symptom onset and CSF collection are variable, which could also possibly affect the result on the proteomic expression profile. Third, the outcomes of patients with VZV meningitis were judged by their conditions at the time of discharge, which could also make the classification inaccurate, following discharge for one or 3 months may be more reliable. Fourth, our study is a retrospective study, VZV meningitis patients with incomplete clinical data were excluded, which may lead to bias in patient selection. Future prospective studies are needed to confirm our results.

## 5 CONCLUSION

Our study suggests that inflammatory response is one of the important pathophysiological mechanisms of VZV CNS infection, and the expression level of IL‐18 protein in CSF might be a potential prognostic indicator that can be used to judge the outcome of VZV meningitis patients.

## AUTHOR CONTRIBUTIONS

**Huili Liu**: Conceptualization; data curation; formal analysis; investigation; writing—original draft. **Jun Wang**: Data curation; formal analysis; investigation; writing—review and editing. **Yan Zhang**: Conceptualization; data curation; investigation. **Jing Gu**: Conceptualization; investigation. **Yu Wang**: Data curation; formal analysis. **Yongxing Yan**: Conceptualization; funding acquisition; resources; writing—review and editing. **Dongli Pan**: Funding acquisition; project administration; writing—review and editing. **Zeyu Sun**: Formal analysis; funding acquisition; project administration; writing—review and editing.

## CONFLICT OF INTEREST STATEMENT

The authors declare no conflict of interest.

## ETHICS STATEMENT

The study protocol was approved by the Ethics Committee of Hangzhou Third People's Hospital (NO: 2021KA013).

## Data Availability

The data that support the findings of this study are available from the corresponding author upon reasonable request.

## References

[1] Furuta Y , Ohtani F , Sawa H , Fukuda S , Inuyama Y . Quantitation of varicella‐zoster virus DNA in patients with Ramsay Hunt syndrome and zoster sine herpete. J Clin Microbiol. 2001;39(8):2856‐2859. 11474003 10.1128/JCM.39.8.2856-2859.2001 PMC88250

[2] Mailles A , De Broucker T , Costanzo P , Martinez‐Almoyna L , Vaillant V , Stahl JP . Long‐term outcome of patients presenting with acute infectious encephalitis of various causes in France. Clin Infect Dis. 2012;54(10):1455‐1464. 22460967 10.1093/cid/cis226

[3] Sundström K , Weibull CE , Söderberg‐Löfdal K , Bergström T , Sparén P , Arnheim‐Dahlström L . Incidence of herpes zoster and associated events including stroke‐a population‐based cohort study. BMC Infect Dis. 2015;15:488. 26520060 10.1186/s12879-015-1170-y PMC4628253

[4] George BP , Schneider EB , Venkatesan A . Encephalitis hospitalization rates and inpatient mortality in the United States, 2000‐2010. PLoS One. 2014;9(9):e104169. 25192177 10.1371/journal.pone.0104169 PMC4156306

[5] Nagel MA , Niemeyer CS , Bubak AN . Central nervous system infections produced by varicella zoster virus. Curr Opin Infect Dis. 2020;33:273‐278. 32332223 10.1097/QCO.0000000000000647 PMC13183292

[6] Grahn A , Studahl M . Varicella‐zoster virus infections of the central nervous system‐prognosis, diagnostics and treatment. J Infect. 2015;71:281‐293. 26073188 10.1016/j.jinf.2015.06.004

[7] Gilden D , White T , Khmeleva N , et al. Prevalence and distribution of VZV in temporal arteries of patients with giant cell arteritis. Neurology. 2015;84:1948‐1955. 25695965 10.1212/WNL.0000000000001409 PMC4433460

[8] Ahmed S , van Zalm P , Rudmann EA , et al. Using CSF proteomics to investigate herpesvirus infections of the central nervous system. Viruses. 2022;14(12):2757. 36560759 10.3390/v14122757 PMC9780940

[9] Tyrberg T , Nilsson S , Blennow K , Zetterberg H , Grahn A . Serum and cerebrospinal fluid neurofilament light chain in patients with central nervous system infections caused by varicella‐zoster virus. J Neurovirol. 2020;26(5):719‐726. 32816287 10.1007/s13365-020-00889-2 PMC7532135

[10] Lind L , Eriksson K , Grahn A . Chemokines and matrix metalloproteinases in cerebrospinal fluid of patients with central nervous system complications caused by varicella‐zoster virus. J Neuroinflammation. 2019;16(1):42. 30777092 10.1186/s12974-019-1428-1 PMC6378740

[11] Grahn A , Hagberg L , Nilsson S , Blennow K , Zetterberg H , Studahl M . Cerebrospinal fluid biomarkers in patients with varicella‐zoster virus CNS infections. J Neurol. 2013;260(7):1813‐1821. 23471614 10.1007/s00415-013-6883-5

[12] Kuhn M , Sühs KW , Akmatov MK , et al. Mass‐spectrometric profiling of cerebrospinal fluid reveals metabolite biomarkers for CNS involvement in varicella zoster virus reactivation. J Neuroinflammation. 2018;15(1):20. 29343258 10.1186/s12974-017-1041-0 PMC5773076

[13] Werner RN , Nikkels AF , Marinović B , et al. European consensus‐based (S2k) guideline on the management of herpes zoster—guided by the European Dermatology Forum (EDF) in cooperation with the European Academy of Dermatology and Venereology (EADV), part 1: diagnosis. JEADV. 2017;31(1):9‐19. 27804172 10.1111/jdv.13995

[14] Nagel MA , Gilden D . Neurological complications of varicella zoster virus reactivation. Curr Opin Neurol. 2014;27(3):356‐360. 24792344 10.1097/WCO.0000000000000092 PMC4189810

[15] Hong HL , Lee EM , Sung H , Kang JK , Lee SA , Choi SH . Clinical features, outcomes, and cerebrospinal fluid findings in adult patients with central nervous system (CNS) infections caused by varicella‐zoster virus: comparison with enterovirus CNS infections: VZV versus EV CNS infection. J Med Virol. 2014;86(12):2049‐2054. 24532558 10.1002/jmv.23902

[16] Jarrin I , Sellier P , Lopes A , et al. Etiologies and management of aseptic meningitis in patients admitted to an internal medicine department. Medicine. 2016;95(2):e2372. 26765411 10.1097/MD.0000000000002372 PMC4718237

[17] Rottenstreich A , Oz ZK , Oren I . Association between viral load of varicella zoster virus in cerebrospinal fluid and the clinical course of central nervous system infection. Diagn Microbiol Infect Dis. 2014;79(2):174‐177. 24666705 10.1016/j.diagmicrobio.2014.02.015

[18] Lee G‐H , Kim J , Kim H‐W , Cho JW . Herpes simplex viruses (1 and 2) and varicella‐zoster virus infections in an adult population with aseptic meningitis or encephalitis: a nine‐year retrospective clinical study. Medicine. 2021;100(46):e27856. 34797322 10.1097/MD.0000000000027856 PMC8601327

[19] Blauwkamp TA , Thair S , Rosen MJ , et al. Analytical and clinical validation of a microbial cell‐free DNA sequencing test for infectious disease. Nat Microbiol. 2019;4(4):663‐674. 30742071 10.1038/s41564-018-0349-6

[20] Kupila L , Vuorinen T , Vainionpaa R , Hukkanen V , Marttila RJ , Kotilainen P . Etiology of aseptic meningitis and encephalitis in an adult population. Neurology. 2006;66(1):75‐80. 16401850 10.1212/01.wnl.0000191407.81333.00

[21] Park SE , Shin K , Song D , et al. Comparison of cerebrospinal fluid cytokine levels in children of enteroviral meningitis with versus without pleocytosis. J Interferon Cytokine Res. 2018;38(8):348‐355. 30052102 10.1089/jir.2018.0002

[22] Lind L , Studahl M , Persson Berg L , Eriksson K . CXCL11 production in cerebrospinal fluid distinguishes herpes simplex meningitis from herpes simplex encephalitis. J Neuroinflammation. 2017;14(1):134. 28693588 10.1186/s12974-017-0907-5 PMC5504603

[23] Maric LS , Lepej SZ , Gorenec L , et al. Chemokines CXCL10, CXCL11, and CXCL13 in acute disseminated encephalomyelitis, non‐polio enterovirus aseptic meningitis, and neuroborreliosis: CXCL10 as initial discriminator in diagnostic algorithm? Neurol Sci. 2018;39(3):471‐479. 29288471 10.1007/s10072-017-3227-8

[24] Sun Z , Li W , Xu J , et al. Proteomic analysis of cerebrospinal fluid in children with acute enterovirus‐associated meningoencephalitis identifies dysregulated host processes and potential biomarkers. J Proteome Res. 2020;19(8):3487‐3498. 32678604 10.1021/acs.jproteome.0c00307

[25] Xu J , Sun Z , Li W , Liu L , Gao F , Pan D . Epidemiological characteristics and cerebrospinal fluid cytokine profiles of enterovirus encephalitis in children in Hangzhou, China. J Med Virol. 2022;94(6):2645‐2652. 34862630 10.1002/jmv.27504

[26] Panato A , Tomasi L , Simon C , et al. Meta‐analysis identifies tumor necrosis factor‐alpha and interleukin‐1 beta as diagnostic biomarkers for bacterial and aseptic meningitis. Curr Neurovasc Res. 2014;11(4):340‐348. 25219657 10.2174/1567202611666140912120940

[27] Sun G , Ota C , Kitaoka S , et al. Elevated serum levels of neutrophil elastase in patients with influenza virus‐associated encephalopathy. J Neurol Sci. 2015;349(1‐2):190‐195. 25626769 10.1016/j.jns.2015.01.017

[28] Ma X , Niu X , Zhao J , et al. Downregulation of Sepina3n aggravated blood‐brain barrier disruption after traumatic brain injury by activating neutrophil elastase in mice. Neuroscience. 2022;503:45‐57. 36089165 10.1016/j.neuroscience.2022.08.023

[29] Rugemalira E , Roine I , Kuligowski J , et al. Protein oxidation biomarkers and myeloperoxidase activation in cerebrospinal fluid in childhood bacterial meningitis. Antioxidants. 2019;8(10):441. 31581487 10.3390/antiox8100441 PMC6826731

[30] Akgun E , Tuzuner MB , Sahin B , et al. Proteins associated with neutrophil degranulation are upregulated in nasopharyngeal swabs from SARS‐CoV‐2 patients. PLoS One. 2020;15(10):e0240012. 33079950 10.1371/journal.pone.0240012 PMC7575075

[31] Christensen JE , de Lemos C , Moos T , Christensen JP , Thomsen AR . CXCL10 is the key ligand for CXCR3 on CD8+ effector T cells involved in immune surveillance of the lymphocytic choriomeningitis virus‐infected central nervous system. J Immunol. 2006;176(7):4235‐4243. 16547260 10.4049/jimmunol.176.7.4235

[32] Suzuki T , Tetsuka S , Ogawa T , Hashimoto R , Okada S , Kato H . An autopsy case of varicella zoster virus encephalitis with multiple brain lesions. Intern Med. 2020;59(13):1643‐1647. 32238719 10.2169/internalmedicine.3417-19 PMC7402971

[33] Schlattner U , Tokarska‐Schlattner M , Wallimann T . Mitochondrial creatine kinase in human health and disease. Biochim Biophys Acta. 2006;1762(2):164‐180. 16236486 10.1016/j.bbadis.2005.09.004

[34] Kerendi H , Rahmati M , Mirnasuri R , Kazemi A . High intensity interval training decreases the expressions of KIF5B and Dynein in Hippocampus of wistar male rats. Gene. 2019;704:8‐14. 30978476 10.1016/j.gene.2019.04.027

[35] Rivera J , Chu PJ , Lewis TL , Arnold DB . The role of Kif5B in axonal localization of Kv1 K(+) channels. Eur J Neurosci. 2007;25(1):136‐146. 17241275 10.1111/j.1460-9568.2006.05277.x

[36] Cromberg LE , Saez TMM , Otero MG , et al. Neuronal KIF5b deletion induces striatum‐dependent locomotor impairments and defects in membrane presentation of dopamine D2 receptors. J Neurochem. 2019;149(3):362‐380. 30664247 10.1111/jnc.14665

[37] Takahashi H , Katayama K , Sohya K , et al. Selective control of inhibitory synapse development by Slitrk3‐PTPδ trans‐synaptic interaction. Nat Neurosci. 2012;15(3):389‐398. 22286174 10.1038/nn.3040 PMC3288805

[38] Weingarten DJ , Shrestha A , Juda‐Nelson K , Kissiwaa SA , Spruston E , Jackman SL . Fast resupply of synaptic vesicles requires synaptotagmin‐3. Nature. 2022;611(7935):320‐325. 36261524 10.1038/s41586-022-05337-1

[39] Masztalerz A , Zeelenberg IS , Wijnands YM , et al. Synaptotagmin 3 deficiency in T cells impairs recycling of the chemokine receptor CXCR4 and thereby inhibits CXCL12 chemokine‐induced migration. J Cell Sci. 2007;120(Pt 2):219‐228. 17179206 10.1242/jcs.03328

[40] Chattopadhyay D , Mukhopadhyay A , Ojha D , Sadhukhan P , Dutta S . Immuno‐metabolic changes in herpes virus infection. Cytokine. 2018;112:52‐62. 29960669 10.1016/j.cyto.2018.06.028

[41] Vastag L , Koyuncu E , Grady SL , Shenk TE , Rabinowitz JD . Divergent effects of human cytomegalovirus and herpes simplex virus‐1 on cellular metabolism. PLoS Pathog. 2011;7:e1002124. 21779165 10.1371/journal.ppat.1002124 PMC3136460

[42] Reed LJ , Lasserson D , Marsden P , Bright P , Stanhope N , Kopelman MD . Correlations of regional cerebral metabolism with memory performance and executive function in patients with herpes encephalitis or frontal lobe lesions. Neuropsychology. 2005;19:555‐565. 16187874 10.1037/0894-4105.19.5.555

[43] Steiner I , Kennedy PG , Pachner AR . The neurotropic herpes viruses: herpes simplex and varicella‐zoster. Lancet Neurol. 2007;6(11):1015‐1028. 17945155 10.1016/S1474-4422(07)70267-3

[44] Persson A , Bergström T , Lindh M , Namvar L , Studahl M . Varicella‐zoster virus CNS disease‐viral load, clinical manifestations and sequels. J Clin Virol. 2009;46(3):249‐253. 19709927 10.1016/j.jcv.2009.07.014

[45] Schiffer JT , Corey L . Rapid host immune response and viral dynamics in herpes simplex virus‐2 infection. Nat Med. 2013;19(3):280‐288. 23467247 10.1038/nm.3103 PMC3981536

[46] Colombo D , Falasca L , Marchioni L , et al. Neuropathology and inflammatory cell characterization in 10 autoptic COVID‐19 brains. Cells. 2021;10(9):2262. 10.3390/cells10092262 34571912 PMC8469459

[47] Sato‐Takeda M , Takasaki I , Takeda K , et al. Major histocompatibility complex haplotype is associated with postherpetic pain in mice. Anesthesiology. 2006;104(5):1063‐1069. 16645460 10.1097/00000542-200605000-00024

